# Laparoscopically assisted versus open colostomy for anorectal malformations: a comparison of postoperative outcomes

**DOI:** 10.1007/s00383-025-06108-5

**Published:** 2025-07-26

**Authors:** G. Axelsson, A. Gunnarsdóttir, T. Wester, A. Löf Granström

**Affiliations:** 1 Department of Women’s and Children’s Health, Karolinska Institutet, Stockholm, Sweden; 2 Unit of Pediatric Surgery, Karolinska University Hospital, Stockholm, Sweden

**Keywords:** Anorectal malformation, Newborn, Colostomy, Laparoscopy, Anorectoplasty, Complications

## Abstract

**Purpose:**

Colostomy is a common procedure in neonates with anorectal malformations (ARM) but carries a risk of complications, prompting the development of a laparoscopically assisted approach to minimize them. This study aimed to compare postoperative outcomes in ARM patients undergoing laparoscopically assisted versus open colostomies.

**Methods:**

Medical records of all newborns with ARM who underwent colostomy at Karolinska University Hospital between 2012 and 2022 were retrospectively reviewed. Patients were grouped based on whether the colostomy was laparoscopy-assisted or fashioned through a limited open incision at the stoma site. Postoperative outcomes, including time to first oral feeding, hospital stay, and complications according to Clavien-Madadi, were compared.

**Results:**

A total of 44 patients were included, of whom 14 underwent laparoscopically assisted colostomy. Among all patients, the median time to first oral feeding was zero days and the median hospital stay was eight days, with no significant group differences. Postoperative complications classified as Clavien–Madadi ≥ IB occurred in 2 patients (14%) in the laparoscopic group and 7 (23%) in the open colostomy group (*p* = 0.6). No significant differences in complication severity were observed.

**Conclusion:**

Laparoscopically assisted colostomy has a similar risk of postoperative complications as open colostomy in patients with ARM.

## Introduction

Congenital anorectal malformations (ARM) comprise a spectrum of rare birth defects, with an estimated prevalence of 1 in 2500 to 1 in 5000 live births [1–3]. These complex conditions typically require specialized reconstructive surgery in early life, often preceded by the creation of a temporary protective colostomy shortly after birth, with definitive repair performed at a later stage [4, 5]. The primary purpose of the colostomy is to divert bowel contents while allowing the child to grow adequately prior to definitive reconstruction.

The open technique remains one of the most used approaches for colostomy creation. Although this traditional method is widely practiced, it carries a substantial complication rate of up to 70%, including stoma prolapse, intestinal obstruction, and wound dehiscence [6]. The traditional open technique has been further refined to involve colostomy creation through a limited incision directly at the stoma site [7]. Laparoscopy-assisted colostomy for colonic diversion in adults was first introduced in 1991, and evidence from adult populations suggests that this approach may improve postoperative outcomes, especially with regard to surgical wound complications [8–10]. The encouraging results from adult studies, combined with the known risks associated with open laparotomy, have led to the adoption of a laparoscopic approach for colostomy creation in children with ARM. This technique provides a possibility of visualization of the abdominal cavity and pelvis, facilitating precise stoma placement in the appropriate bowel segment while minimizing the risk of colonic loop twisting during formation [11, 12]. Several case series and a systematic review have demonstrated the laparoscopic approach to be a safe and effective technique for colostomy creation in patients with ARM [11–17]. However, most of these studies have primarily focused on the feasibility of laparoscopy, and direct comparative evidence regarding its efficacy in reducing postoperative complications, as compared with the open approach, remains limited.

The aim of this study was to compare postoperative outcomes in children with ARM undergoing laparoscopically assisted versus open colostomy.

## Methods

### Study design

This was a retrospective cohort study.

### Study setting and participants

All patients with ARM who had undergone colostomy creation at the Unit of Pediatric Surgery at Karolinska University Hospital between 2012 and 2022 were eligible for the study. Patients were categorized into two groups based on whether the colostomy was laparoscopy-assisted or fashioned through a limited open incision at the intended stoma site. The method of stoma creation was determined by the operating surgical team.

### Data sources and variables

Patient characteristics and clinical variables were recorded retrospectively from the medical records. Clinical variables analyzed in this study included the type of colostomy, type of ARM, operative time, time to first oral feeding, length of hospital stay, postoperative complications within 30 days, postoperative complication beyond 30 days, and time to colostomy closure. Postoperative complications were classified according to the Clavien-Madadi classification [18]. The operative time (minutes) was defined as the interval from the initial skin incision to the placement of the final skin suture.

### Statistical methods

Statistical analyses were performed using R (version 4.2.1) within RStudio (version 2022.07.01). Data wrangling was performed with the *dplyr* package, and tables were created using *gtsummary*, unless otherwise specified [19]. Categorical variables are presented as frequencies and percentages, while continuous variables are reported as median with interquartile range (Q1, Q3). For comparative analyses of categorical variables, the chi-square test was applied when appropriate, and Fisher’s exact test was used when the assumptions of the chi-square test were not met. For continuous variables, comparisons between two independent groups were made using the Wilcoxon rank sum test. A *p*-value ≤ 0.05 was considered statistically significant.

### Ethical considerations

The study was conducted in accordance with the principles of the Declaration of Helsinki and its later amendments [20]. The study was approved by the Swedish Ethical Review Authority.

## Results

### Patient characteristics

A total of 44 patients were included in the study, of whom 14 underwent laparoscopically assisted colostomy and 30 underwent colostomy fashioned through a limited open incision. The characteristics of the study population are presented in Table 1. Sex distribution and gestational age were comparable between the two groups, with no statistically significant differences. Overall, 77% of patients had at least one associated congenital anomaly. The most common malformations were cardiovascular malformations (32.2%), followed by urogenital (21.8%), musculoskeletal (21.8%), nervous system (10.3%), other gastrointestinal (8.0%), bronchopulmonary (4.6%), and oropharyngeal malformations (1.1%). Chromosomal abnormalities were identified in four patients, all of whom had trisomy 21.

**Table 1:** Patient Characteristics

Characteristic	Overall *N* = 44	Laparoscopically assisted colostomy creation		*p*-value^1^
		No *N* = 30	Yes *N* = 14	
Female, n (%)	19 (43%)	11 (37%)	8 (57%)	0.2
Gestational Week, Median (Q_1_, Q_3_)	38 (37, 40)	38 (37, 40)	39 (37, 40)	0.8
Birth Weight, Median (Q_1_, Q_3_)	2,899 (2,515, 3,589)	2,894 (2,500, 3,585)	3,173 (2,720, 3,600)	0.4
Type of Anorectal Malformation, n (%)				0.3
Imperforate anus without fistula	5 (12%)	5 (18%)	0 (0%)	
Perineal fistula	8 (19%)	6 (21%)	2 (14%)	
Persistent cloaca	1 (2.4%)	1 (3.6%)	0 (0%)	
Rectourethral bulbar fistula	8 (19%)	6 (21%)	2 (14%)	
Rectourethral prostatic fistula	4 (9.5%)	2 (7.1%)	2 (14%)	
Vestibular fistula	16 (38%)	8 (29%)	8 (57%)	
Associated Congenital Anomalies, n (%)	34 (77%)	22 (73%)	12 (86%)	0.5
Chromosomal Aberrations, n (%)	4 (10%)	3 (12%)	1 (7.7%)	> 0.9

^1^Pearson’s Chi-squared test; Wilcoxon rank sum test; Fisher’s exact test

### Surgical outcomes

The median age at the time of colostomy creation was comparable between the two groups (two days). Postoperative complications classified as Clavien-Madadi ≥ IB occurred in two patients (14%) with laparoscopically assisted colostomy and in seven (23%) patients with open colostomy (*p* = 0.7). There were no statistically significant differences in postoperative complications, either within or beyond 30 days after colostomy creation, between the two groups. Additionally, no significant differences were observed in the severity of postoperative complications between the two groups. Postoperative outcomes following colostomy creation are summarized in Table 2.

**Table 2:** Summary of Results from Colostomy Creation; Open Colostomy vs. Laparoscopically Assisted

Characteristic	Overall *N* = 44	Laparoscopically assisted colostomy creation		p-value^1^
		No *N* = 30	Yes *N* = 14	
Age at colostomy (Days), Median (Q_1_, Q_3_)	2.00 (1.00, 2.04)	2.00 (1.00, 2.00)	2.00 (2.00, 3.00)	0.11
Location of Colostomy, n (%)				
Sigmoid	44 (100%)	30 (100%)	14 (100%)	
Type of Colostomy, n (%)				> 0.9
Divided	1 (2.3%)	1 (3.3%)	0 (0%)	
Loop	43 (98%)	29 (97%)	14 (100%)	
Fascia Suture, n (%)	31 (70%)	24 (80%)	7 (50%)	0.074
Time to Per Oral Feeding (Days), Median (Q_1_, Q_3_)	0.00 (0.00, 1.00)	0.00 (0.00, 1.00)	0.00 (0.00, 0.00)	0.3
Length of Stay (Days), Median (Q_1_, Q_3_)	8 (5, 19)	8 (6, 21)	6 (5, 19)	0.6
Post-operative Complication (within 30 Days), n (%)	9 (20%)	7 (23%)	2 (14%)	0.7
Complications Clavien-Madadi (within 30 Days), n (%)				0.6
IB	2 (22%)	2 (29%)	0 (0%)	
II	3 (33%)	2 (29%)	1 (50%)	
IIIA	1 (11%)	1 (14%)	0 (0%)	
IIIB	1 (11%)	0 (0%)	1 (50%)	
IV	2 (22%)	2 (29%)	0 (0%)	
Postoperative Complication (> 30 Days), n (%)	6 (19%)	5 (23%)	1 (10%)	0.6
Postoperative Complication (Overall), n (%)	14 (32%)	11 (37%)	3 (21%)	0.5

^1^Wilcoxon rank sum test, Fisher’s exact test

Postoperative complications within 30 days of colostomy creation included one case of necrotizing enterocolitis, one case of sepsis, two cases of colostomy prolapse, and single cases of skin excoriation, wound infection, subileus, paralytic ileus, and tracheal mucus plug. Postoperative complications occurring beyond 30 days included one death, five cases of colostomy prolapse, and one case of intestinal obstruction. Notably, five out of six cases of colostomy prolapse occurred in patients who had undergone open colostomy creation (*p* = 0.65). Of the six cases of colostomy prolapse, four arose from the distal limb and two from the proximal limb of the stoma.

## Discussion

Colostomy creation is a fundamental component of managing ARM, with the primary purpose to allow the child to grow adequately prior to definitive reconstruction. The stoma creation may either be performed through an open technique or laparoscopically assisted. In this study, we retrospectively analyzed data from patients with ARM who underwent colostomy creation, using either a laparoscopically assisted or traditional open surgical technique. No statistically significant differences in postoperative outcomes were observed between those who received a laparoscopically assisted versus an open colostomy. Postoperative complications classified as Clavien-Madadi ≥ IB occurred in 2 patients (14%) in the laparoscopic group and 7 (23%) in the open colostomy group (*p* = 0.6). No significant differences in complication severity were observed.

In this study, we observed an overall postoperative complication rate of 21% in patients who underwent laparoscopically assisted colostomy, compared with 37% in those who underwent colostomy via an open approach. Although this difference was not statistically significant, the trend is in line with findings reported by Ramirez-Amoros et al., who demonstrated a statistically significant reduction in complications with laparoscopically assisted colostomy (9.1%) compared to open colostomy (48.3%) [17]. It is important to note, however, that in that study, all laparoscopic procedures involved loop sigmoid colostomies, whereas the open procedures were exclusively divided sigmoid colostomies. In contrast, nearly all colostomies in our study–regardless of surgical technique–were loop sigmoid colostomies, reducing variability related to stoma type and potentially influencing complication rates. Despite these differences, the overall rates of postoperative complications observed in our cohort are broadly comparable to those reported in prior studies. Saxena et al. reported a 17% complication rate in a case series of six ARM patients undergoing laparoscopically assisted colostomy [15], while Liem and Quynh reported no postoperative complications in their series of 39 newborns [13]. Similarly, Gine et al. observed no postoperative complications in their series of six ARM patients treated with the laparoscopic approach [14]. These studies reinforce the need for larger cohorts to more accurately assess the postoperative safety and efficacy of laparoscopically assisted colostomy in this patient population. Regardless, our findings align with existing literature with postoperative complication rates comparable to–or potentially lower than–those associated with the open approach [11–15, 17].

Regarding the severity of postoperative complications following colostomy creation, there were no significant differences in the severity of postoperative complications between the two groups. The types of postoperative complications observed in this study were consistent with those reported in previous studies on colostomy creation in patients with ARM [12, 15, 21]. However, it is worth noting that one case of necrotizing enterocolitis was observed postoperatively in our cohort. This patient had a complex clinical profile, including ARM, long-gap esophageal atresia with a tracheoesophageal fistula, and a congenital cardiac defect requiring prolonged intensive care. Given the patient’s multimorbidity and extended intensive care unit stay, this event is more plausibly attributable to the overall clinical complexity rather than a direct postoperative complication of colostomy creation.

Compared with the open procedure, the laparoscopic colostomy technique offers comparable benefits and postoperative outcomes while additionally affording enhanced visualization of the intra-abdominal and genitourinary anatomy, enabling precise stoma placement in the appropriate bowel segment and reducing the risk of colonic loop twisting during formation [11, 12]. This improved visualization and precise stoma placement allow for careful preservation of sufficient distal bowel length, which is crucial for the subsequent anorectal reconstruction. Notably, five out of the six cases of stoma prolapse in this study occurred in patients who underwent open colostomy creation. Although the difference did not reach statistical significance, this pattern may suggest a potential benefit of the laparoscopic approach in minimizing stoma-related complications, particularly prolapse. The aforementioned enhanced visualization and control afforded by laparoscopy could contribute to more optimal stoma positioning and tension-free bowel configuration, thereby reducing the risk of prolapse. Future prospective studies with larger sample sizes are warranted to further investigate this association.

## Limitations

A key limitation of this study lies in its retrospective nature, which may limit the ability to control for confounding variables and introduce the possibility of selection bias. Future research in the form of a prospective, preferably randomized, trial is necessary to more rigorously evaluate the advantages and potential disadvantages of laparoscopically assisted colostomy compared to the conventional open approach.

## Conclusion

Laparoscopically assisted colostomy carries a comparable risk of postoperative complications to the conventional open approach in patients with ARM. Given its additional advantages–such as improved intraoperative visualization and more precise stoma placement–it represents a viable and potentially beneficial option in the surgical management of these patients.

## Data Availability

No datasets were generated or analysed during the current study.
